# Evaporation of liquid nanofilms: A minireview

**DOI:** 10.1063/5.0082191

**Published:** 2022-02-08

**Authors:** Kaixuan Zhang, Wei Fang, Cunjing Lv, Xi-Qiao Feng

**Affiliations:** Institute of Biomechanics and Medical Engineering, AML, Department of Engineering Mechanics, Tsinghua University, Beijing 100084, China

## Abstract

Evaporation of virus-loaded droplets and liquid nanofilms plays a significant role in the pandemic of COVID-19. The evaporation mechanism of liquid nanofilms has attracted much attention in recent decades. In this minireview, we first introduce the relationship between the evaporation process of liquid nanofilms and the pandemic of COVID-19. Then, we briefly provide the frontiers of liquid droplet/nanofilm evaporation on solid surfaces. In addition, we discuss the potential application of machine learning in liquid nanofilm evaporation studies, which is expected to be helpful to build up a more accurate molecular model and to investigate the evaporation mechanism of liquid nanofilms on solid surfaces.

## INTRODUCTION

The outbreak of the COVID-19 pandemic has caused great loss of human health and lives worldwide.^1–3^ The transmission of COVID-19 through the food cold chain is mainly through infected individuals who generate saliva droplets with SARS-CoV-2 onto the surface of food packaging by speaking, coughing, sneezing, and breathing, and then the SARS-CoV-2 enters new hosts during subsequent stages such as transportation and distribution, thus leading to infection.^4–6^ Addressing this problem requires not only enhanced protection of individuals but also effective active intervention or even elimination of SARS-CoV-2 adhering to surfaces. This requires a better understanding of the mechanisms of evaporative diffusion of droplets containing SARS-CoV-2 on solid surfaces such as food packaging.

**FIG. 1.**
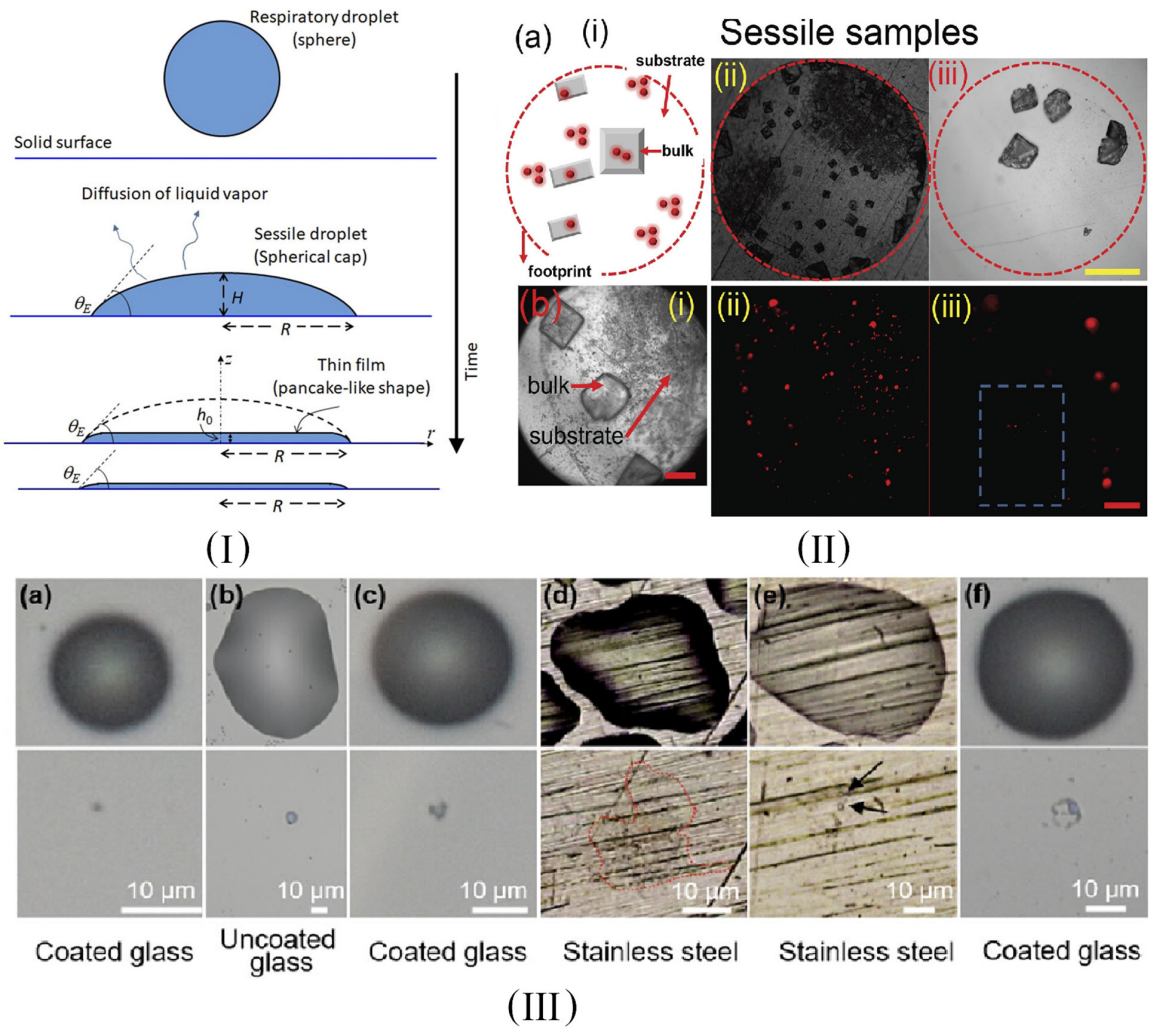
(I) Schematic of evaporation of the thin film that forms in the later stages of droplet evaporation. Reproduced with permission from R. Bhardwaj and A. Agrawal, Phys. Fluids 32, 111706 (2020). Copyright 2020 AIP Publishing.^13^ (II) Evaporation of a sessile liquid droplet/film. Reproduced with permission from S. Basu *et al.*, Phys. Fluids 32, 123317 (2020). Copyright 2020 AIP Publishing.^15^ (III) Evaporation of a liquid drop/film on different kinds of solid substrates. Reproduced with permission from Z. He *et al.*, Phys. Fluids 33, 013309 (2021). Copyright (2021) AIP Publishing.^14^

In fact, virus-containing droplets evaporate once they have been separated from the infected person. Larger sized drops generally settle rapidly onto nearby solid surfaces to continue evaporation, while smaller ones evaporate rapidly into droplet nuclei and float in the air, which then spread to greater distances with the surrounding airflow.^7–9^ During this process, the *in vitro* survival of the viruses is largely dependent on the content of water in the droplet. The reduction of water due to evaporation significantly affects the viral activity. Recent theoretical and experimental studies^10–12^ have shown that in the nucleus of a droplet suspended in air, the SARS-CoV-2 could survive for roughly three hours, while on some solid surfaces, the new coronavirus may survive for several days and remain infectious for some time, and even longer on cold surfaces. The reason is that when droplets evaporate on a solid surface, most of the water evaporates within a few minutes, leaving only a small amount of nanometers' thick liquid film. However, this part of the nanofilm evaporates extremely slowly and is able to provide the necessary environment for the survival of the SARS-CoV-2 for a longer period of time.^13–15^ Figure 1(I) provides a schematic for the evaporation process from a liquid droplet to a thin film. The corresponding experimental results show that the evaporation process of liquid drop/nanofilms can be really different in various environments, as shown in Fig. 1(III). The details about the experimental setup are given in the related references. In that case, the transmission of COVID-19 through the cold chain is extremely insidious and continuously dangerous and is also the focus and difficulty of epidemic prevention and control at the current stage.^2^ Therefore, studying the evaporative mechanism of liquid nanofilms on solid surfaces and analyzing the related factors that influence the heat transfer efficiency are crucial to accelerate the evaporation process and lead to virus inactivation due to lack of water, thus avoiding the transmission of SARS-CoV-2 through the food cold chain.

Nanofilms exhibit a layer-by-layer evaporation process, which is characterized by liquid-phase atoms breaking away from the liquid–gas interface at the upper surface of the liquid film and then becoming gas-phase atoms.^16^ According to the classical theory of liquid nanofilm evaporation, the heat transfer from the surface of the solid wall to the liquid–gas interface needs to overcome the thermal resistance of the solid–liquid interface and the thermal conductivity of the liquid film. The absorption of heat by the liquid phase atoms at the liquid–gas interface into the gas phase atoms requires overcoming the liquid–gas interface thermal resistance. Therefore, the analysis of the heat transfer process at the solid–liquid interface and the morphological changes at the liquid–gas interface during evaporation is particularly important for understanding the evaporation heat transfer mechanism of liquid nanofilms.

It has been shown that both enhanced wettability and roughness of the solid surface can promote the heat transfer performance at the solid–liquid interface.^17^ Increasing surface wettability can enhance the heat transfer process by improving the heat transfer coupling characteristics between the solid and the liquid, while increasing surface roughness can significantly increase the effective contact area between the solid and liquid phases, thus enhancing the heat transfer process. Song and Chen^18^ indicated that both of these approaches improve the interfacial heat transfer by effectively reducing the thermal resistance of the solid–liquid interface. However, the understanding of the molecular mechanism of the thermal resistance at the solid–liquid interface remains largely unexplored, so further investigation is needed. Due to the difficulty of capturing the liquid–gas interface during the nanoscale evaporation process, previous studies on the dynamic changes of the liquid–gas interface morphology are quite scarce. In the study of the liquid–gas interface, Wang *et al.*^19^ proposed that the liquid–gas interface at the molecular scale is not a flat surface, but a curved partitioned interface that rises and falls with time, and that the interface thickness is the width of the rise and fall region. At present, there are very few studies that focus on the molecular mechanism of the liquid–gas interface morphology. Moreover, the definition of the thickness of the liquid–gas interface at the nanoscale is still controversial in different studies. The reason is that most of the existing literature works are crude in capturing the liquid–gas interface morphology, so large errors are introduced in the research methods, which leads to different perceptions of the liquid–gas interface thickness. Therefore, a more accurate description of the dynamic change of the liquid–gas interface during evaporation is needed, which will enable us to analyze the molecular mechanisms involved in order to deeply understand the morphological change mechanism of the liquid–gas interface.

Thus, by studying the evaporative mechanism of liquid nanofilms on a solid surface and optimizing the design of surface parameters to enhance the heat transfer performance, thus accelerating the evaporation process of liquid nanofilms, it is of great guidance to effectively suppress the cold chain transmission of SARS-CoV-2.

## EVAPORATION OF LIQUID NANOFILMS ON SOLID SURFACES

For the study of the heat transfer process of liquid nanofilm evaporation on solid surfaces, molecular dynamics, as a bottom–up particle-based method, has been widely used to describe the energy transfer process in nanoscale multiphase flow systems by simulating the interactions between molecules. The effects of nanofilm thickness, surface temperature, surface material, wettability, and roughness on the heat transfer characteristics, such as evaporation rate, heat transfer rate, and heat flow density, in the evaporation process are considered.^20^

The evaporation process occurs at the liquid–gas interface, and the variation of the liquid film thickness not only directly affects the heat transfer inside the liquid film but also affects the heat transfer at the solid–liquid interface. Therefore, the liquid film thickness is very important for the evaporation heat transfer process. Cao *et al.*^21^ pointed out that the increase in the liquid film thickness brings a significant increase in the thermal resistance of heat conductivity of the liquid film, which weakens the evaporation process. By simulating the evaporative heat transfer process of argon liquid nanofilms of different thicknesses on a metal surface, Wang *et al.*^22^ indicated that there is a critical thickness accounting for the transition between the liquid film evaporation and the boiling behavior, and the boiling behavior is dominant when the liquid film thickness is larger than this critical thickness; on the contrary, the evaporation behavior is dominant when the liquid film thickness is less than this critical thickness. In addition, as the liquid film thickness increases, the time to reach the equilibrium state of evaporation becomes longer.^23^ Liang *et al.*^24^ indicated that the thickness variation of the liquid film also affects the thermal resistance at the solid–liquid interface, which affects the evaporative heat transfer process. However, the condition is only highlighted when the liquid film thickness is less than 1.5 nm, and the thermal resistance at the solid–liquid interface increases rapidly with the liquid film thickness; when it is greater than the thickness of four layers of argon atoms, the increase in the thermal resistance at the solid–liquid interface becomes slow and the efficiency of heat transfer gradually converges to a stable constant. For different liquid film thicknesses, the changes of the solid–liquid interface thermal resistance as well as the thermal resistance of heat conductivity of the liquid film indicate that the density of water molecule vibration near the interface has changed significantly. In this process, how the dynamic structure of water molecules evolves remains unclear. Therefore, to clearly reveal the underlying physical mechanism, a precise description of the kinetic evolution of the close contact layer at the solid–liquid interface and the water molecules in the nearby homogeneous phase is needed.

The influence of the surface temperature on the evaporation behavior is significant. Hens *et al.*^25^ found that argon nanofilms exhibit layer-by-layer evaporation on smooth surfaces at 90 and 150 K, while burst boiling occurs on surfaces at 250 K. By simulating the heat transfer process at the solid–liquid interface at a mesoscopic scale, the applicant^26^ reproduced the thermal capillarity phenomenon of the surface temperature gradient driving microdroplet motion. At the nanoscale, the surface temperature gradient may also affect the evaporation behavior of liquid nanofilms. At present, studies involving the effect of the surface temperature on the phase change heat transfer process of liquid nanofilms have mainly focused on the analysis at higher surface temperatures, and the analysis of the effect of lower surface temperatures on the evaporation process is lacking. In the transmission of the COVID-19 epidemic through the food cold chain, the evaporation heat transfer process plays a major role in the low temperature environment. In this regard, research on the evaporation mechanism of liquid nanofilms at lower surface temperatures is essential.

Previous studies on the influence of surface materials on the evaporative heat transfer process of liquid nanofilms have mainly focused on the study of face-centered cubic structure metal materials, which are mostly common metals such as platinum and aluminum.^25^ Most of the research methods were used to realize the evaporation process on the surface of different materials by changing the lattice constant, the depth of the potential trap between the solid–solid and solid–liquid, and the characteristic length, using molecular dynamics simulations.^27^ Hasan *et al.*^27^ found that the heat flow density and evaporation rate on the surface of platinum and silver are stronger than those on the surface of aluminum. Recent studies on the evaporation process of liquid nanofilms containing virus on solid surfaces have shown that the liquid nanofilms may exist for several days on plastic surfaces at low temperatures, while it only exists for a few hours on copper surfaces.^13^ This difference in evaporation duration is crucial for the survival of SARS-CoV-2, so it is necessary to investigate the mechanism of enhanced heat transfer by evaporation of liquid nanofilms on different material surfaces.

Differences in surface wettability can significantly affect the heat transfer performance at the solid–liquid interface. By studying the evaporation process of liquid nanofilms on hybrid surfaces consist of hydrophilic/hydrophobic triangular regions, Wan *et al.*^28^ found that the evaporation rate of the liquid phase in the hydrophobic region is proportional to the contact line length. Han *et al.*^29^ found that the thermal resistance of the solid–liquid interface plays an important role in accurately predicting the heat flux of evaporation at the nanoscale when they studied the evaporation of liquid nanofilms near the solid–liquid–vapor three-phase contact line. Lu's group^30^ also noticed the critical influence of the tightly adsorbed layer near the three-phase contact line on the heat and mass transfer process when studying the evaporation of droplets. Wang *et al.*^31^ proposed that the enhancement of surface wettability would reduce the thermal resistance between the solid surface and the liquid adsorption layer but meanwhile might enhance the thermal resistance between the liquid adsorption layer and the free liquid layer, and the strength of this effect is related to the distribution of water molecules near the interface. Most of the current studies focus on the influence of the surface wettability on the heat transfer performance, while the underlying physics has not been explored from a molecular point of view. A study of the molecular mechanism in evaporative heat transfer is necessary to deeply understand the enhanced mechanism of evaporative heat transfer of liquid nanofilms on solid surfaces.

Research on the effect of surface roughness on the evaporation process of liquid nanofilms is generally divided into two aspects: one is to study the strengthening effect of a specific rough structure on the evaporative heat transfer; the other one is to study the effect of a rough structure on the thermal resistance of the solid–liquid interface. Sun and He^32^ suggested that the strong solid–liquid coupling between the nanostructure and the liquid nanofilm is the main reason for the enhanced heat transfer through the study of the evaporative heat transfer process of liquid nanofilms on the surface of a rectangular nanostructure. Li *et al.*^33^ indicated that the distribution of the nanostructure and the relative position to the liquid film determine whether the heat transfer process can be enhanced. By studying the liquid film evaporation heat transfer on the surface of concentric circular nanostructures, Gao *et al.*^34^ found that the concentric circular structure can promote the diffusion of liquid phase atoms to the upper regime of low potential energy, thus promoting the transformation of the wetting process and finally achieving enhanced evaporation heat transfer. In contrast, the wavy rough structure hinders the diffusion of the liquid-phase atoms, but at the same time, the increase in the liquid-phase atoms in the recessed region promotes the movement of liquid-phase atoms to the depressed middle, thus enhancing the collision of atoms between liquid phases and improving the evaporation rate.^35^ In contrast, the two-dimensional rectangular recesses enhance the heat transfer process by reducing the thermal resistance at the solid–liquid interface.^36^ Wu *et al.*^37^ established the Cantor set fractal rough surface by the fractal theory and analyzed the liquid nanofilm evaporation behavior and the variation of the liquid nanofilm thickness in the non-evaporating layer. The results showed that the liquid nanofilm evaporation response on the Cantor set fractal rough surface was faster, and the thickness of the non-evaporative layer was higher, thus improving and enhancing the evaporative heat transfer process of the liquid nanofilms. Luo *et al.*^38,39^ investigated the effect of the self-assembled monolayer (SAM) length on the heat transfer process, and the results showed that SAMs with interacting lengths can effectively enhance the solid–liquid interface heat transfer process, which is similar to the fin heat exchanger in macroscopic heat exchangers, increasing the effective heat transfer area and, thus, enhancing the heat transfer efficiency. In addition, there are related studies on surfaces with textures such as cones, cylinders, spheres, triangles, fingers, T-shapes, and concave/convex hemispheres.^40–42^ It is evident that parameters, such as the shape and the rough structures, play a decisive role in enhancing the heat transfer efficiency. However, most of the current studies have analyzed a particular rough structure, and a unified description of the mechanisms influencing the rough structure is still lacking. This requires an in-depth analysis of the molecular mechanisms that affect the thermal resistance at the solid–liquid interface and the evaporative heat transfer process.

The solid–liquid interface thermal resistance plays an important role in the evaporation process of liquid nanofilms. The improvement of the surface roughness can effectively reduce the thermal resistance of the solid–liquid interface and, thus, enhance the evaporation heat transfer process. Jiang's team^43^ found that structural parameters, such as the contact area and the contact thickness between two contact surfaces connected by rectangular nanostructures, affect the solid–liquid interface thermal resistance, which increases with decreasing contact area of nanostructures and increases with increasing nano-thickness at constant temperature. Wang *et al.*^44^ investigated the solid–liquid interface. The increase in the surface roughness effectively reduced the solid–liquid interface temperature difference and significantly decreased the solid–liquid interface thermal resistance by comparing with the smooth surface. Huang *et al.*^38^ showed that the use of an organic monolayer can also effectively reduce the solid–liquid interface thermal resistance by increasing the solid–liquid heat transfer area and increasing the solid–liquid interaction. By controlling the same wettability and equivalent area ratio, He *et al.*^45^ found that different shapes of rough structural units (e.g., triangles, rectangles, and sinusoidal curves) were in turn less effective in enhancing interfacial heat transfer. This indicates that the rough element shape has some influence on the interfacial heat transfer, and the change in the interfacial heat transfer strength is mainly caused by the change in the thermal resistance at the solid–liquid interface. By analyzing the liquid phase zone number density, temperature distribution, and interfacial thermal resistance length, Wang's team^19^ also confirmed that the presence of the surface roughness reduced the solid–liquid interfacial thermal resistance, thus strengthening the energy transfer at the solid–liquid interface. The current research on the thermal resistance of the solid–liquid interface on rough surfaces mainly focuses on simple comparisons of the trends of the heat transfer performance of different types of rough surfaces, parameters such as heat flux are considered, and it is still worthy of further study on how the rough structure affects the thermal resistance of the solid–liquid interface and the mechanism of its enhanced effect on the evaporative heat transfer process of liquid nanofilms.

## MACHINE LEARNING AND MOLECULAR SIMULATION

The booming development of machine learning techniques has enabled it to solve problems that are difficult to handle by traditional methods and, thus, has received wide attention in various fields such as drug design^46^ and protein structure analysis.^47^ The introduction of machine learning into scientific computing can achieve the objectives of improving computational efficiency, filling in missing data, and mining new physical mechanisms.^48,49^ Computer simulation studies of nanoscale liquid film evaporation heat transfer usually use molecular dynamics simulation methods. In molecular dynamics, the correctness of simulation results basically depends on the accuracy of the force field in the model, while the speed of calculation is closely related to the complexity of the built force field and the scale of the system. Therefore, it has been an important topic of molecular simulation research to construct molecular potentials that can respond to the complex inter-atomic interactions with high accuracy while reducing the computational complexity.^50^

Most of the traditional force fields in molecular dynamics are established from manual summaries of experimental data, which are empirical or semi-empirical methods. This approach is less accurate, the construction model is not uniform and standardized enough, and it is difficult to achieve all the various properties of the material, so by now hundreds of new force field models appear every year. For example, the water molecule model contains the TIP3P model, and the improved TIP4P and TIP5P models, as well as the SPC and mW models.^51^ The potential energy functions (also called potential surfaces) obtained by using the more underlying quantum chemistry or first-principles calculations, although they can guarantee the computational accuracy and generality of molecular simulations, also bring a staggering computational cost. Its computational effort is in a seventh-order relationship with the computational scale, which basically limits the system size to the dynamical solution of tens of molecules; the improved submodel named coupled cluster singles and doubles (CCSD) theory reduces the computational effort to a sixth-order; and the density function theory (DFT), which is now widely adopted, reduces the computational effort to a third-order. Although considerable progress has been made in reducing the computational effort from seventh to third power, such a large computational effort still severely limits its application in molecular simulations.

Machine learning has been developing rapidly in recent years with tremendous progress in image processing and speech recognition, and the combination with other scientific fields is being explored. The idea of combining machine learning with high-precision molecular simulations to achieve accelerated computation has been carried out by some top teams internationally.^52–54^ This new idea of introducing the capability of machine learning into the field of molecular simulation, if realized, will be an important progress in the field of molecular simulation and promotes the fundamental research on the heat and mass transfer in multiphase flows and its related applications.

In nanoscale multiphase fluid flows, the reproduction of complex flow phenomena with heat and mass transfer relies on highly accurate molecular simulations but meanwhile requires large scale systems to show the full picture of the whole problem such as nucleation and bubble growth as well as heterogeneous chemical reactions.^55,56^ This puts traditional force field-based and quantum chemistry-based molecular simulations in a difficult situation concerning simulation accuracy and computational efficiency, respectively. Therefore, it is necessary to construct novel molecular potentials with both high accuracy and low computational complexity. In the evaporative process of liquid nanofilms on solid surfaces, the heat transfer performance of the solid–liquid interface is closely related to the laminar distribution pattern of water molecules near the close contact layer of the interface. Among them, the molecular mechanism of action regarding the surface wettability, nano-rough structure, surface temperature, and liquid nanofilm thickness affecting the thermal resistance of the solid–liquid interface is still unclear.^28^ On the other hand, the process of water molecular dynamics at the liquid–gas interface and its mechanism analysis during evaporation of the liquid nanofilms also need to be further investigated.^57^

To accurately describe the transition process of water molecules from the liquid phase to the gas phase near the liquid–gas interface, there is, therefore, a great necessity to include the dynamic processes of such large span systems as solid–liquid, liquid–gas interfaces, and homogeneous bulk phase water in the simulation. The DFT method, which meets the accuracy requirements, can currently only simulate very small amounts of water molecules at the solid–liquid or liquid–gas interfaces.^58,59^ One of the difficulties is that the method is difficult to simulate the dynamics of such large systems. Moreover, decreasing the accuracy or reducing the size of the simulation may limit the comprehensive understanding of the problem. Therefore, by introducing machine learning into molecular simulation, the molecular features in the system are extracted by learning a large amount of high-precision physical information; while ensuring that the novel molecular potentials have high accuracy, the computational effort can be greatly reduced and the computational efficiency improved in order to further explore the physical mechanisms behind the complex flow phenomena underneath the pandemic of COVID-19.^60–64^

## CONCLUSIONS

In summary, there is a serious lack of understanding of the mechanism of evaporation of liquid nanofilms on solid surfaces, especially on the molecular mechanisms of the thermal resistance at the solid–liquid interface and morphological changes at the liquid–gas interface, which need to be studied in depth. The DFT method with first-principles accuracy is limited by the large computational volume, and it is difficult to simulate the dynamics of large-scale systems including the solid–liquid and liquid–gas interfaces and homogeneous phase water; while for the molecular model based on traditional force fields, it is difficult to describe the dynamics of water molecules during evaporation with sufficient accuracy. One alternative way is to introduce machine learning into molecular simulation to construct a molecular potential model with high accuracy and low computational complexity. The model will be used to simulate the evaporative heat transfer process of liquid nanofilms on solid surfaces. Then, we can use it to investigate the effects of surface roughness, temperature, wettability, and liquid nanofilm thickness on the evaporative heat transfer performance and reveal the mechanism of enhanced evaporative heat transfer of liquid nanofilms on solid surfaces. This can be expected to enhance the evaporative heat transfer efficiency of liquid nanofilms by studying the evaporative heat transfer strengthening mechanism of liquid nanofilms and optimizing the design of surface parameters, so as to provide a theoretical basis for preventing the spread of the COVID-19 epidemic through the food cold chain.

## Data Availability

The data that support the findings of this study are available within the article.
